# Sympathetic Innervation Regulates Osteocyte‐Mediated Cortical Bone Resorption during Lactation

**DOI:** 10.1002/advs.202207602

**Published:** 2023-04-26

**Authors:** Qiaoyue Guo, Ningrong Chen, Cheng Qian, Cheng Qi, Kathleen Noller, Mei Wan, Xiaonan Liu, Weixin Zhang, Patrick Cahan, Xu Cao

**Affiliations:** ^1^ Department of Orthopedic Surgery Johns Hopkins University School of Medicine Baltimore MD 21205 USA; ^2^ Department of Biomedical Engineering Johns Hopkins University School of Medicine Baltimore MD 21205 USA

**Keywords:** cortical bone remodeling, lactation, osteocytes, osteocytic bone resorption, sympathetic nerves

## Abstract

Bone undergoes constant remodeling by osteoclast bone resorption coupled with osteoblast bone formation at the bone surface. A third major cell type in the bone is osteocytes, which are embedded in the matrix, are well‐connected to the lacunar network, and are believed to act as mechanical sensors. Here, it is reported that sympathetic innervation directly regulates lacunar osteocyte‐mediated bone resorption inside cortical bone. It is found that sympathetic activity is elevated in different mouse models of bone loss, including lactation, ovariectomy, and glucocorticoid treatment. Further, during lactation elevated sympathetic outflow induces netrin‐1 expression by osteocytes to further promote sympathetic nerve sprouting in the cortical bone endosteum in a feed‐forward loop. Depletion of tyrosine hydroxylase‐positive (TH^+^) sympathetic nerves ameliorated osteocyte‐mediated perilacunar bone resorption in lactating mice. Moreover, norepinephrine activates *β*‐adrenergic receptor 2 (Adrb2) signaling to promote secretion of extracellular vesicles (EVs) containing bone‐degrading enzymes for perilacunar bone resorption and inhibit osteoblast differentiation. Importantly, osteocyte‐specific deletion of Adrb2 or treatment with a *β*‐blocker results in lower bone resorption in lactating mice. Together, these findings show that the sympathetic nervous system promotes osteocyte‐driven bone loss during lactation, likely as an adaptive response to the increased energy and mineral demands of the nursing mother.

## Graphical abstract

During lactation, norepinephrine (NE) levels increase in cortical bone. In response, osteocytes increase Ca^2+^ signaling to promote the secretion of bone resorptive extracellular vesicles and cathepsin K (Ctsk) to degrade the perilacunar area bone matrix. In parallel, NE signaling promotes netrin‐1 expression, thus leading to the increased innervation of TH^+^ sympathetic nerves toward cortical bone in a feedback loop manner.

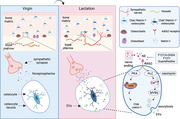

## 1 Introduction

Bone is one of the largest organs, accounting for 20% of our body weight, and it provides mechanical support, as well as being a key reserve for calcium and other minerals.^[^ ^1^ ^]^ Bone mass peaks in humans in the third decade of life, but then declines continuously, with an increased risk for osteoporosis and major skeletal disorders occurring from that point on.^[^ ^2^, ^3^ ^]^ In adults, bone undergoes constant remodeling via osteoclast‐mediated bone resorption that is exquisitely coupled to osteoblast‐directed bone formation to facilitate proper calcium metabolism, but also to repair the microcracks that appear daily due to the forces of gravity and mechanical stress.^[^ ^4^, ^5^ ^]^ However, such bone remodeling is primarily conducted at the bone surface,^[^ ^6^ ^]^ which only represents a small portion of the total bone mass.

A third major cell type within the bone is osteocytes, which are embedded in the bone matrix and account for 80% of the total bone cell population.^[^ ^7^ ^]^ These long‐lived dendritic cells are interconnected through their dendrites within the mineralized bone matrix in the lacunar‐canalicular system (LCS), and are believed to have mechanosensitive properties, and thus act like a sensory system.^[^ ^8^, ^9^ ^]^ During lactation, osteocytes can mediate bone resorption via the LCS to provide calcium and other minerals for breast milk.^[^ ^10^ ^]^ In addition, the cortical bone is mobilized to compensate for the depletion of calcium and other mineral during aging, resulting in the gradual development of porosity and trabeculation of this region of the bone.^[^ ^11^, ^12^ ^]^ Interestingly, mechanical stimulation activates Ca^2+^ oscillations in osteocytes that lead to the release of extracellular vesicles (EVs), and blocking Ca^2+^ signaling significantly attenuates bone adaptation to mechanical loading.^[^ ^13^ ^]^ This observation suggests that systemic regulation of bone metabolism by Ca^2+^‐mediated signaling occurs via the dendritic network of osteocytes.

Skeletal interoception regulates bone homeostasis, with hypothalamus‐induced sympathetic activity inhibiting bone formation and promoting bone resorption.^[^ ^14^, ^15^, ^16^ ^]^ Notably, tyrosine hydroxylase (TH)‐derived norepinephrine (NE) is the primary neurotransmitter released from sympathetic nerve endings that activates the *β*2 adrenergic receptor (Adrb2) in osteoblast lineage cells.^[^ ^14^, ^17^, ^18^ ^]^ Conditional Adrb2 knockout mice demonstrate that osteoblasts are the primary cells for sympathetic activity regulation of bone formation.^[^ ^18^ ^]^ Along these lines, we recently showed that the central nervous system (CNS) regulates osteoblastic bone formation and thus bone density through prostaglandin E2 (PGE2)‐mediated bone sensory nerve activation that is relayed back to the hypothalamus to tone down sympathetic outflow.^[^ ^19^ ^]^ More specifically, we found that PGE2 is secreted by osteoblasts in response to mechanical loading of bone, which leads to the activation of PGE2 receptor 4 (EP4) in the hypothalamus to stimulate phosphorylation of CREB and the downregulation of TH expression that in turn leads to reduced sympathetic activity. This reduction in sympathetic outflow allows for the greater osteoblastic differentiation of mesenchymal stem/stromal cells (MSCs) and subsequent increased bone formation.^[^ ^20^ ^]^ Interestingly, sympathetic nerve‐derived NE promotes osteocyte secretion of neuropeptides.^[^ ^21^, ^22^, ^23^ ^]^ Therefore, it is possible that the CNS could systemically regulate both osteoblast bone formation and osteocyte‐mediated endocrine activity through the LCS by modulating sympathetic nerve activity.

Osteocyte‐mediated perilacunar bone resorption has been reported.^[^ ^24^, ^25^ ^]^ Recent studies have shown that osteocytes express genes related to bone resorption, such as tartrate‐resistant acid phosphatase (TRAP), cathepsin K (Ctsk), and matrix metalloproteinases, to resorb bone matrix and minerals in the lacunar‐canalicular areas.^[^ ^10^, ^26^, ^27^ ^]^ It is not clear how osteocytes directly promote bone resorption as osteocytes do not form a sealing zone to form resorption pits, similar to OCs. The lacunae are enlarged and bone mass is decreased in mice during lactation, as well as in mice treated with glucocorticoids or those who undergo ovariectomy (OVX).^[^ ^25^ ^]^ Moreover, vesicle‐like structures have been detected in the osteocyte lacunar‐canalicular network.^[^ ^28^ ^]^ Mechanical loading stimulates EV production by activation of Ca^2+^ signaling in osteocytes.^[^ ^13^ ^]^ Importantly, osteocytes communicate with each other and with cells on the bone surface through their dendritic connections to the lacunar‐canalicular network.^[^ ^9^ ^]^ Hence, the activation of Ca^2+^ signaling in osteocytes by sympathetic activity could potentially broadly transmit the nerve‐provoked signals through the dendritic lacunar‐canalicular network to regulate bone metabolism.

Women lose 4%–6% of their bone mass during breastfeeding, while mice lose almost one‐third of their bone volume during 2 weeks of lactation.^[^ ^29^, ^30^ ^]^ Interestingly, the lost bone mass is restored after weaning in many mammalian species.^[^ ^25^, ^31^ ^]^ Therefore, mouse lactation is an ideal model to study the dynamic nature of bone mass regulation, including a possible role for the sympathetic nervous system in the regulation of osteocyte‐mediated bone metabolism, as well as the underlying molecular mechanisms involved in osteocyte‐mediated bone resorption.

In this study, we sought to investigate skeletal interoception in the regulation of osteocyte‐mediated bone resorption using mouse lactation as a model of bone loss. Specifically, we investigated whether there was an alteration in sympathetic activity and thus NE levels in different models of bone loss in mice as modulation of NE and sympathetic activity is the descending pathway of skeletal interoception. We found that NE and sympathetic innervation were increased during lactation, as well as after OVX and after treatment with an exogenous glucocorticoid, which reduced osteoblast differentiation and promoted EV release by the osteocytes for perilacunar bone resorption.

## 2 Results

### 2.1 Sympathetic Activity is Increased in Lactating Mice, Glucocorticoid‐Induced, and OVX Mice

To examine if sympathetic nerve activity regulates osteocyte‐induced perilacunar bone resorption, we examined the NE content and changes in cortical bone content of the tibia in mice either after 12 days of lactation, 4 weeks of exogenous glucocorticoid treatment with methylprednisolone or 4 weeks post‐OVX (**Figure** **1**a–c). We collected the cortical bone of the tibia after the study periods indicated above in test and control mice for each of the three models, and each set of samples was homogenized into bone chips and analyzed for NE levels by ELISA (Figure 1d). We found that the levels of NE were significantly higher in the bone chips from all three mouse models of bone loss, compared to their controls (Figure 1e–g). To verify whether the NE level will fall back after weaning since lacunae size will recover after weaning.^[^ ^10^ ^]^ We collected tibia cortical bone chips from 14 days post‐lactation mice, and the NE level was measured by ELISA. We found that the NE content was significantly decreased in weaning mice compared to lactating mice (Figure S1a, Supporting Information). We further performed TRAP and immunofluorescence staining of the femur sections of the lactating mice and the virgin controls as a role for osteocytes in the resorption of cortical bone during lactation has been reported.^[^ ^10^ ^]^ We found that the percentage of both TRAP^+^ and Ctsk^+^ osteocytes in cortical bone were significantly greater (Figure 1h–k), while the numbers of osteocalcin (OCN)^+^ osteoblasts were significantly decreased in lactating mice relative to virgin mice (Figure 1l,m).

Figure 1Sympathetic activity is increased in lactating mice, as well as those treated with glucocorticoid or after OVX. a–c) Schematic diagrams illustrating the three mouse models used in the study: lactation (a), glucocorticoid treatment (methylprednisolone, MPS) (b), and OVX (c). d) Schematic diagram showing the procedure for the isolation of the bone chips which were used in ELISA. e–g) Norepinephrine levels of isolated cortical bone chips in lactation (e), glucocorticoid treatment (f), and OVX (g) mouse models compared to their corresponding control groups. n = 9 in the lactation model, and n = 5 in the glucocorticoid treatment and OVX models. h, i) Tartrate‐resistant acid phosphatase (TRAP) staining (purple) (h) and Ctsk staining (green) (i) in cortical bone (femur mid‐shaft) of virgin and lactating mice. Boxed areas are shown at higher magnification in corresponding panels to the right. Scale bars, 40 (left panels), 20 µm (right panels). j,k) Quantification of the percentage of TRAP^+^ osteocytes (j) and the percentage of Ctsk^+^ osteocytes (k) in total osteocytes. n = 7. l,m) Immunofluorescence staining of femoral sections by osteocalcin (OCN) (red) (l) in virgin and lactating mice, and quantification of average OCN^+^ cell number per mm^2^ of tissue area (m). Scale bars, 200 µm (left panels), 100 µm (right panels). n = 4. n–t) Representative micro‐computed tomography (µCT) images (n) and quantitative analysis of total porosity (Po.tot) (o), close porosity (Po.cl) (p), open porosity (Po.op) (q), tissue mineral density (TMD) (r), cortical thickness (Cor.Th) (s) of femoral cortical bone and trabecular bone fraction (BV/TV) (t) in virgin and lactating mice. Scale bar, 1 mm. n = 9. u–w) Alcian Blue staining (u) and quantification of osteocyte lacuna area (v) and osteocyte lacuna perimeter (w) in cortical bone of virgin and lactating mice. n = 7. Data are presented as mean ± standard error of the mean (SEM). Statistical significance was determined by two‐tailed Student's *t*‐test.

**Figure:**
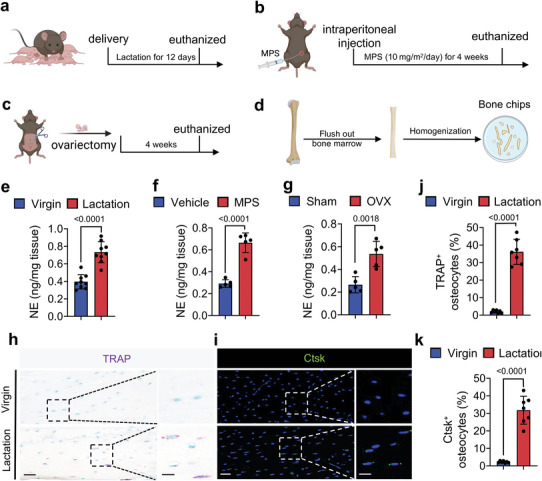

**Figure:**
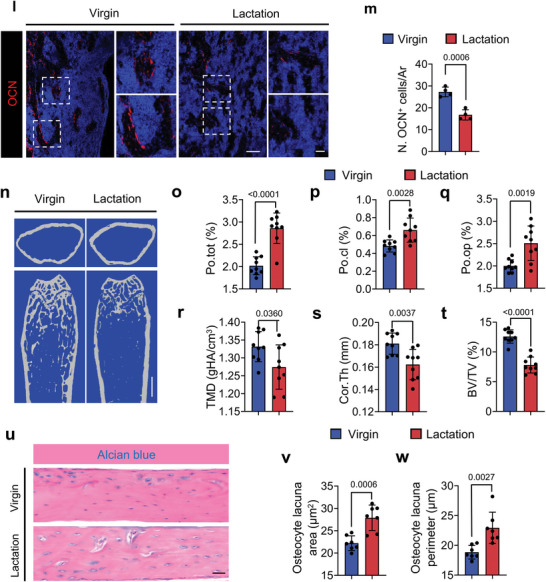

Next, we harvested the femurs from the lactating mice and virgin controls, and we measured their bone density parameters by microcomputed tomography (µCT) (Figure 1n). We found that the percentage of total porosity (Po.tot), percentage of close porosity (Po.cl), and percentage of open porosity (Po.op) were significantly higher, while the tissue mineral density (TMD), the cortical thickness (Cor.Th), and the trabecular bone volume (BV/TV) were significantly lower, in the lactating mice compared to the virgin mice (Figure 1o–t). Histological analysis of femur sections also showed that the osteocyte perilacunar resorption areas were significantly greater in lactating mice compared to virgin mice, with a distinctly greater osteocyte lacunar area and osteocyte lacunar perimeter in the lactating mice (Figure 1u–w). These observations suggest that an increase in sympathetic activity is associated with both cortical and trabecular bone loss.

### 2.2 Lactation‐Induced Osteocyte Perilacunar Resorption is Ameliorated by a *β*‐Blocker

**Figure 2:**
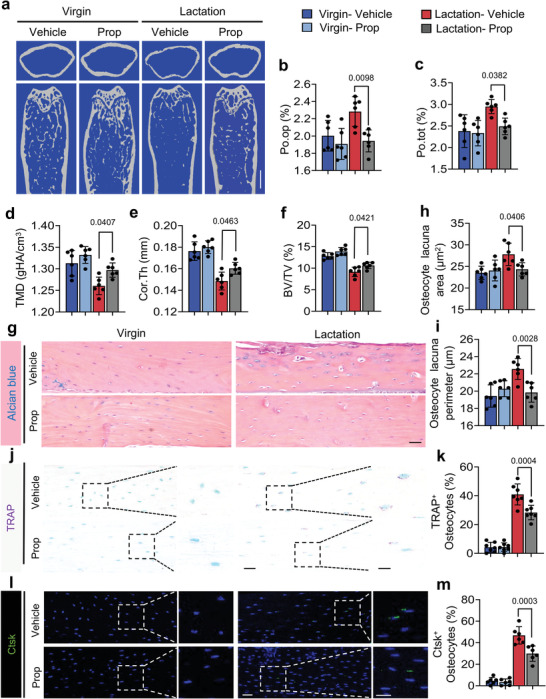
Lactation‐induced osteocyte perilacunar resorption is ameliorated by a *β*‐blocker. a–f) Representative micro‐computed tomography (µCT) images (a) and quantitative analysis of open porosity (Po.op) (b), total porosity (Po.tot) (c), tissue mineral density (TMD) (d), and cortical thickness (Cor.Th) (e) of femoral cortical bone and trabecular bone fraction (BV/TV) (f) in different treatment groups. Scale bar, 1 mm. n = 6. g–i) Alcian Blue staining (g) and quantification of osteocyte lacuna area (h) and osteocyte lacuna perimeter (i) of cortical bone in different treatment groups. n = 6. j,k) Representative TRAP staining images of cortical bone (femur mid‐shaft) (j) and quantification of TRAP^+^ osteocytes on femur cortical bone (k) in virgin and lactating mice with different treatments. Boxed areas are shown at a higher magnification in corresponding panels to the right. Scale bar, 40 µm (left panels), 20 µm (right panels). n = 7. l,m) Immunofluorescence staining of Ctsk (green) at mid‐shaft of the femur (l) and quantification of Ctsk^+^ osteocytes in the cortical bone (m) in virgin and lactating mice with different treatments. Boxed areas are shown at a higher magnification in corresponding panels to the right. Scale bar, 40 µm (left panels), 20 µm (right panels). n = 6. Data are presented as mean ± SEM. Statistical significance was determined by two‐way repeated measures analysis of variance (ANOVA) with Tukey post hoc test.

Sympathetic nerve activity regulates target cells by releasing NE from nerve endings.^[^ ^17^ ^]^ To examine whether an increase in sympathetic activity promotes osteocyte resorption of cortical bone, we treated lactating mice with propranolol (Prop), a non‐selective *β*‐blocker. The percentage of Po.op and Po.tot of femoral cortical bone in lactating mice was significantly lower after Prop treatment based on µCT analysis relative to the vehicle group (**Figure** **2**a–c). The TMD, Cor.Th, and BV/TV were significantly greater after Prop treatment in the lactating mice compared to the vehicle‐treated lactating mice. (Figure 2d–f).

Consistent with the µCT results, the osteocyte lacunar area and osteocyte lacunar perimeter were significantly lower after Prop treatment in the lactating mice compared to the vehicle‐treated lactating mice (Figure 2g–i). As expected, the percentages of TRAP^+^ and Ctsk^+^ osteocytes in cortical bone were also significantly lower in lactating mice with Prop treatment compared to those treated with vehicle (Figure 2j–m). These results indicate that elevation of sympathetic nerve activity stimulates bone resorption through *β*‐adrenergic receptor signaling, which is correlated with changes in the osteocyte population.

### 2.3 Osteocyte‐Specific Deletion of Adrb2 Reduces Osteocyte Bone Resorption in Lactating Mice

**Figure 3:**
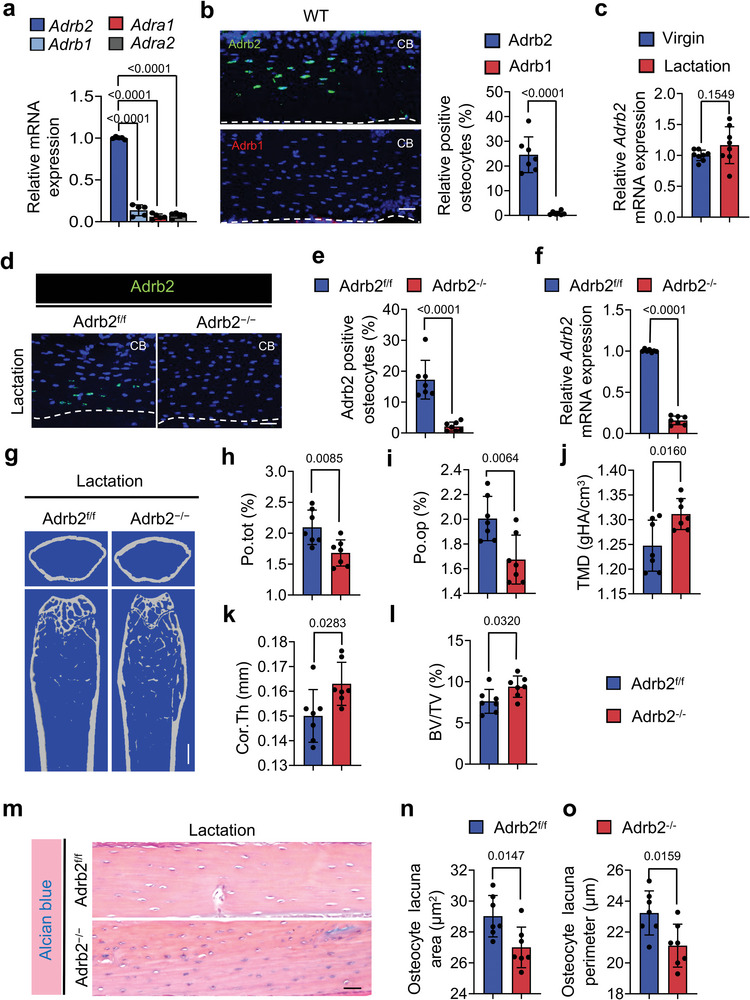
Knockout of Adrb2 in Dmp1^+^ osteocytes attenuates lactation‐induced osteocytic resorption in cortical bone. a) mRNA expression of Adrb2, Adrb1, Adra1, and Adra2 in femoral cortical bone of 3‐month‐old WT mice measured via RT‐qPCR. n = 5. b) Representative images of immunofluorescence staining (left) and quantitative analysis (right) of Adrb2^+^ (green) and Adrb1^+^ (red) osteocytes in cortical bone of 3‐month‐old WT mice. Scale bar, 40 µm. n = 7. c) mRNA expression of Adrb2 in cortical bone of WT virgin mice and lactating mice measured by RT‐qPCR. n = 8. d,e) Immunofluorescence staining (d) and quantification (e) of the percentage of Adrb2 positive osteocytes (green) in cortical bone of Adrb2^flox/flox^ and Adrb2*^−/−^* mice. Scale bar, 40 µm. n = 7. f) mRNA expression of Adrb2 in cortical bone of Adrb2^flox/flox^ and Adrb2*^−/−^* mice after 12 days of lactation measured via RT‐qPCR. n = 7. g–l) Representative micro‐computed tomography (µCT) images (g) and quantitative analysis of total porosity (Po.tot) (h), open porosity (Po.op) (i), tissue mineral density (TMD) (j), and cortical thickness (Cor.Th) (k) of femoral cortical bone and trabecular bone fraction (BV/TV) (l) in Adrb2^flox/flox^ and Adrb2*^−/−^* mice after 12 days of lactation. Scale bar, 1 mm. n = 7. m–o) Alcian Blue staining (m) and quantification of osteocyte lacuna area (n) and osteocyte lacuna perimeter (o) of cortical bone in Adrb2^flox/flox^ and Adrb2*^−/−^* mice after 12 days of lactation. n = 7. Data are presented as mean ± SEM. Statistical significance was determined by one‐way ANOVA with Dunnet post hoc test and two‐tailed Student's *t*‐test.

NE activates the signaling of *α*‐ or *β*‐adrenergic receptors to regulate downstream gene expression.^[^ ^17^ ^]^ We found that the gene Adrb2, which encodes for *β*‐adrenergic receptor 2, was the primary adrenergic receptor expressed in the osteocytes of cortical bone, as measured by RT‐qPCR (**Figure** **3**a). Immunostaining of femoral sections from wildtype (WT) mice further confirmed that Adrb2 is highly expressed in osteocytes compared to Adrb1 (Figure 3b). To ensure lactation doesn't cause any changes in the expression level of Adrb2 in further study, we also assessed its expression in virgin and lactating mice, and no statistically significant difference was found between them (Figure 3c). To validate that it is the activation of Adrb2 signaling that induces osteocyte bone resorption downstream of sympathetic signaling, we crossed Adrb2^flox/flox^ (Adrb2^f/f^) mice with *Dmp1‐cre* mice to generate *Dmp1‐cre*:: Adrb2^flox/flox^ (Adrb2*^−/−^)* mice, in which Adrb2 is selectively deleted in osteocytes. RT‐qPCR and Immunofluorescent staining of Adrb2 confirmed the efficiency of Adrb2 deletion in the osteocytes (Figure 3d–f). The percentage of Po.tot and Po.op were both significantly decreased, while TMD, Cor.Th, and BV/TV were all significantly increased in lactating Adrb2*^−/−^* mice relative to their lactating floxed littermates (Figure 3g–l). Alcian blue staining confirmed that the osteocyte lacuna area and perimeter were both lower in lactating Adrb2^−/−^ mice relative to lactating floxed control littermates (Figure 3m–o). These data show that *β*‐adrenergic receptor 2 signaling in osteocytes regulates bone resorption.

### 2.4 NE‐Activated Adrb2 Signaling Regulates Osteocyte Perilacunar Resorption by inducing CREB Phosphorylation

**Figure 4:**
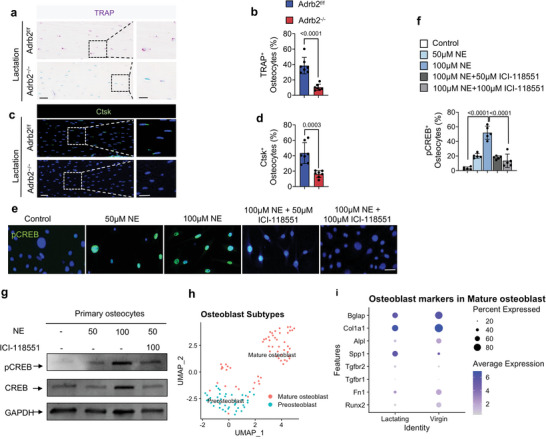
Lactation‐induced osteocyte perilacunar resorption occurs through NE‐mediated Adrb2 signaling. a,b) Representative images of TRAP staining at femur mid‐shaft (a) and quantification of TRAP^+^ osteocytes (b) in Adrb2^flox/flox^ and Adrb2*^−/−^* mice after 12 days of lactation. Boxed areas are shown at a higher magnification in corresponding panels to the right. Scale bar, 40 µm (left panels), 20 µm (right panels). n = 7. c,d) Representative images of immunofluorescence staining of Ctsk (green) at femur mid‐shaft (c) and quantification of Ctsk^+^ osteocytes (d) in Adrb2^flox/flox^ and Adrb2*^−/−^* mice after 12 days of lactation. Boxed areas are shown at a higher magnification in corresponding panels to the right. Scale bar, 40 µm (left panels), 20 µm (right panels). n = 7. e,f) Immunofluorescence staining of pCREB (green) in primary osteocytes treated with different concentrations of NE, NE+ICI‐118551, and control group (f), and quantification of the percentage of pCREB^+^ osteocytes (e). DAPI stains nuclear blue. Scale bar, 20 µm. n = 5. g) Representative immunoblots of pCREB and CREB in primary osteocytes treated with different concentrations of NE, NE+ICI‐118551, and control group. n = 5. h) Bioinformatics analysis of scRNA‐seq of isolated cortical bone cells from virgin and lactating mice with clustering of osteoblasts in (Figure S2d, Supporting Information). i) Dot plot of osteoblast marker genes in osteoblast populations (cluster 11) of virgin and lactating mice. Data are presented as mean ± SEM. Statistical significance was determined by unpaired, two‐tailed Student's *t*‐test (b, d) and one‐way ANOVA with Dunnet post hoc test (f).

Next, we histologically stained femoral sections from lactating Adrb2*^−/−^* mice and their floxed controls and found that the percentage of TRAP^+^ or Ctsk^+^ osteocytes in the cortical bone was significantly lower in the knockout mice compared to the floxed mice (**Figure** **4**a–d). To test whether NE activates Adrb2 signaling to induce CREB phosphorylation (pCREB), primary osteocytes were isolated and cultured with two different concentrations of NE with or without ICI‐118551, a selective Adrb2 inhibitor, for 30 min. By immunostaining, we found that pCREB was induced in the primary osteocytes with 50 µm NE and even further so with 100 µm NE, and this induction was blocked by co‐treatment with ICI‐118551 (Figure 4e,f). Furthermore, we found that pCREB was markedly induced by 100 µm NE in primary osteocytes but blocked by co‐treatment with ICI‐118551 (Figure 4g). Collectively, our results indicate that NE signaling via Adrb2 promotes the maintenance of osteocytes in the cortical bone and that it activates pCREB signaling.

We also examined the changes in osteoblastic cells during lactation as a decrease of sympathetic tone signals promotes osteoblast differentiation.^[^ ^15^, ^18^ ^]^ We prepared scRNA‐seq libraries from single‐cell suspensions of femoral and tibial cortical bone from lactating or virgin mice. Quality control was performed to exclude potential doublets and low‐quality libraries. We performed clustering to identify the major transcriptional states in our data.^[^ ^32^ ^]^ There were 12 clusters that were identified by annotation based on known osteoblast markers and differential gene expression, and SingleCellNet^[^ ^33^ ^]^ was used to classify individual cells based on a well‐annotated reference dataset^[^ ^34^ ^]^ (Figure S2a, Supporting Information). Analysis of marker gene expression in clusters revealed that osteoblastic cells were primarily in cluster 11(Figure S2b,c, Supporting Information), which was clearly divided into preosteoblast and mature osteoblast subclusters (Figure S2d, Supporting Information, Figure 4h). Further analysis of these two subclusters regarding their expression of osteoblast marker genes showed that the expression of osteoblastic cell differentiation maker genes decreased in lactating mice (Figure 4i). The results suggest that an increase in sympathetic activity during lactation also reduces osteoblast differentiation while osteocyte bone resorption is promoted.

### 2.5 Sympathetic Activity Stimulates Secretion of EVs from Osteocytes

Osteocytes are known to secrete EVs under mechanical stimuli.^[^ ^35^ ^]^ To explore the potential mechanism by which sympathetic activity regulates osteocyte activity, primary osteocytes were cultured and exposed to exosome‐depleted medium with 50 µm, 100 µm, 500 µm, or 1 mm NE or vehicle for 3 days (**Figure** **5**a). Flow cytometry results demonstrate the purity of the primary osteocytes obtained (Figure 5b). Transmission electron microscope and analysis exhibit particles with EV morphology and size, suggesting osteocytes secrete EVs in response to NE stimuli (Figure 5c,d). Osteocyte EV protein lysates were analyzed by Western blot (Figure 5e). We found that the proteins Alix, Tsg101, and Ctsk were elevated significantly in the EVs and that their levels peaked with 100 µm NE treatment (Figure 5e,f), consistent with the increase in CREB phosphorylation induced by NE at this dose (Figure 4e–g). By immunodetection we also further validated the increase of Tsg101 expression in osteocytes upon NE treatment, with a peak at 100 µm (Figure 5g).

Figure 5NE stimulates the release of EVs by osteocytes. a) Schematic diagram illustrating the procedure for isolating EVs from primary osteocytes. b) Examination of the purity of the isolated primary osteocytes obtained in (a) by flow cytometry. c,d) Representative images showing EVs visualized by transmission electron microscopy (TEM) (c) and quantification of percentage of different sized EVs (d). e,f) Immunoblots of Alix, Tsg101, and Ctsk from protein lysates of the isolated EVs (e) and quantification of expression levels after normalized to GAPDH (f). g) Immunofluorescence staining of Tsg101 (green) in primary osteocytes after NE stimulation with different concentrations. DAPI stains nuclear blue. Scale bar, 20 µm. h) Calcium concentration over time in primary osteocytes after NE stimulation. i,j) Immunoblots for Alix, Tsg101, and Ctsk in protein lysates from EVs after stimulation with 100 µm of NE with/without addition of 10 mm of neomycin (i), and quantification of Alix, Tsg101, and Ctsk expression levels (j) after being normalized to GAPDH. Data are presented as mean ± SEM. Statistical significance was determined by one‐way ANOVA with Dunnet post hoc test (f, j).

**Figure:**
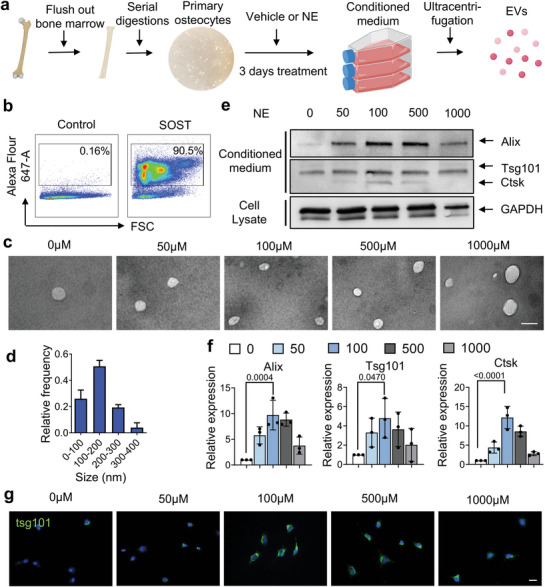

**Figure:**
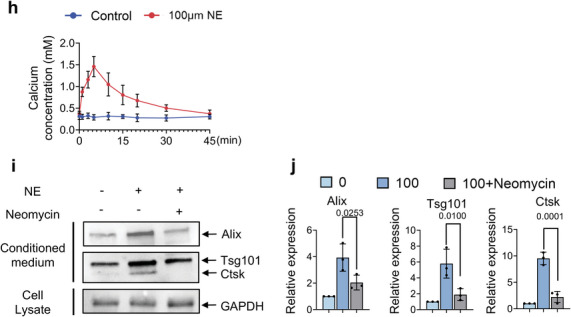

Given the prior findings showing a role for Ca^2+^ oscillations in the release of EVs by osteocytes,^[^ ^13^ ^]^ we next investigated the role of calcium in NE‐stimulated EV release by primary osteocytes. First, we found that intracellular Ca^2+^ concentrations increased robustly during the first 5 min of 100 µm NE treatment, which progressively diminished over the next 40 min (Figure 5h). We then tested whether inhibition of Ca^2+^ signaling could blunt NE‐induced EV release. Primary osteocytes were treated with exosome‐depleted medium with different doses of NE, vehicle, or a combination of the calcium signaling inhibitor neomycin. By western blot analysis of the protein lysates from the stimulated osteocytes we found that 100 µm NE could potently increase the levels of Alix, Tsg101, and Ctsk in the EVs, and this was efficiently blocked by co‐treatment with neomycin (Figure 5i,j). Together, these results show that NE stimulates EV release by osteocytes in a Ca^2+^‐dependent manner. Furthermore, the released EVs contain bone regulatory proteins, including Ctsk, which may mediate osteocytic bone resorption.

### 2.6 Sympathetic Innervation Increased During Lactation

We next examined how sympathetic innervation and activity were induced during lactation. We first measured NE concentrations at different time points during lactation. We found that the NE levels in cortical bone were significantly and progressively increased during lactation in mice over the course of 12 days compared to baseline (**Figure** **6**a). Consistent with the elevation of NE levels, the percentage of TRAP^+^ osteocytes also progressively increased in quantity and in distance from the endosteum to the middle of cortical bone during 12 days of lactation, whereas no change in either parameter occurred in the age‐matched virgin control mice (Figure 6b–e). Further, inhibition of Adrb2 signaling with ICI‐118551 in lactating mice markedly blunted the increase in TRAP^+^ osteocytes and their distance from the endosteum compared to lactating mice treated with vehicle (Figure 6c–e). Likewise, the increase in TRAP^+^ osteocytes and their distance from the endosteum in cortical bone was also markedly blunted in Adrb2*^−/−^* lactating mice relative to lactating Adrb2^f/f^ littermates (Figure 6f–h). These observations suggest that sympathetic innervation is induced during lactation, leading to a progressive increase of TRAP^+^ osteocytes to the periosteal cortical bone surface.

Figure 6Adrb2 signaling promotes the netrin‐1^+^ osteocyte number in the endosteum and increases sympathetic tone during lactation. a) NE levels in mice's cortical bone chips at different time points during lactation as measured by ELISA. b) Schematic diagram illustrating the anatomy of cortical bone. c–e) Distribution of TRAP^+^ osteocytes in middle‐shaft of the femur in virgin and lactating mice treated with vehicle (upper panel) or ICI‐18551 (bottom panel) (c) and quantification of the percentage of TRAP^+^ osteocytes (d) and their distance to the endosteum (e). n = 5. f–h) Distribution of TRAP^+^ osteocytes in middle‐shaft of the femur in Adrb2^flox/flox^ and Adrb2*^−/−^* mice after 12 days of lactating (f) and quantification of the percentage of TRAP^+^ osteocytes (g) and their distance to the endosteum (h). n = 7. i,j) Immunofluorescence staining of tyrosine hydroxylase (TH, red) (i) and quantification of the density of TH^+^ nerves (red) in the endosteum area (j) in femoral bone marrow. E, endosteum; BM, bone marrow. n = 6. k–m) Double‐immunofluorescence images of Ctsk (red) and Netrin‐1 (green) in femoral bone sections from virgin and lactating mice treated with vehicle or ICI‐18551 (k) and quantification of the percentage of Netrin‐1^+^ osteocytes (l) and Netrin‐1^+^/ Ctsk^+^ osteocytes in the femur mid‐shaft (m), respectively. Scale bar, 20 µm. n–p) Double‐immunofluorescence images of Ctsk (red) and netrin‐1 (green) in femoral bone sections from virgin and lactating Adrb2^flox/flox^/ Adrb2^−/^ *^−^* mice (n) and quantification of the percentage of Netrin‐1^+^ osteocytes (o) and Netrin‐1^+^/ Ctsk^+^ osteocytes in the femur mid‐shaft (p), respectively. Scale bar, 20 µm. n = 7. q) mRNA expression of *Ntn1* by RT‐qPCR in tibial cortical bone of Adrb2^flox/flox^ and Adrb2^−/−^ virgin mice. n = 5. Data are presented as mean ± SEM. Statistical significance was determined by one‐way ANOVA with Dunnet post hoc test (a, j), two‐way repeated measures ANOVA with Tukey post hoc test (l, m, o, p), and two‐tailed Student's *t*‐test (q).

**Figure:**
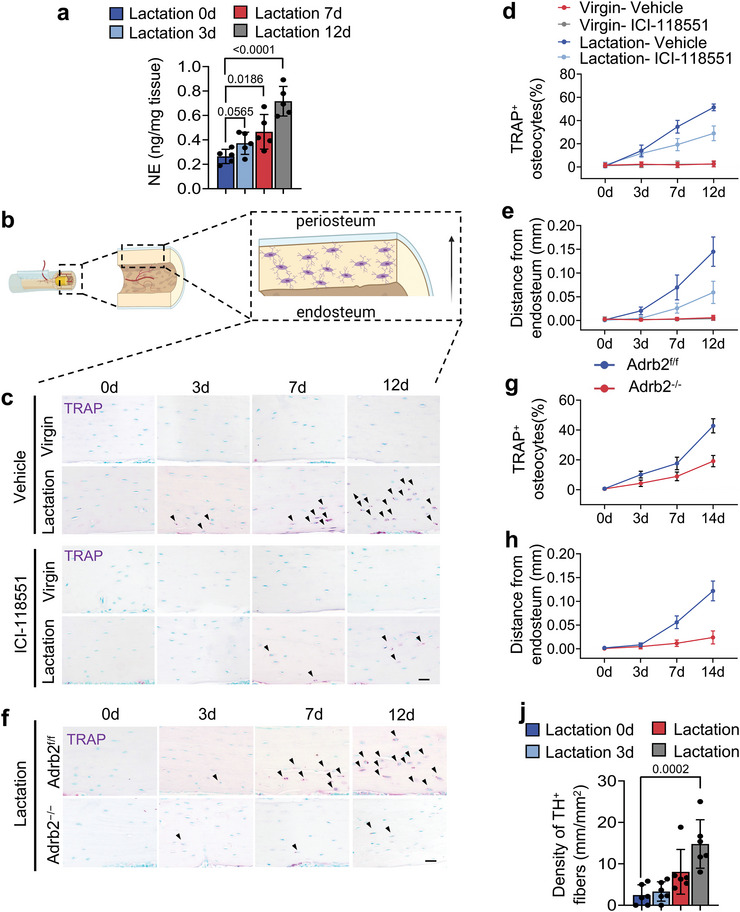

**Figure:**
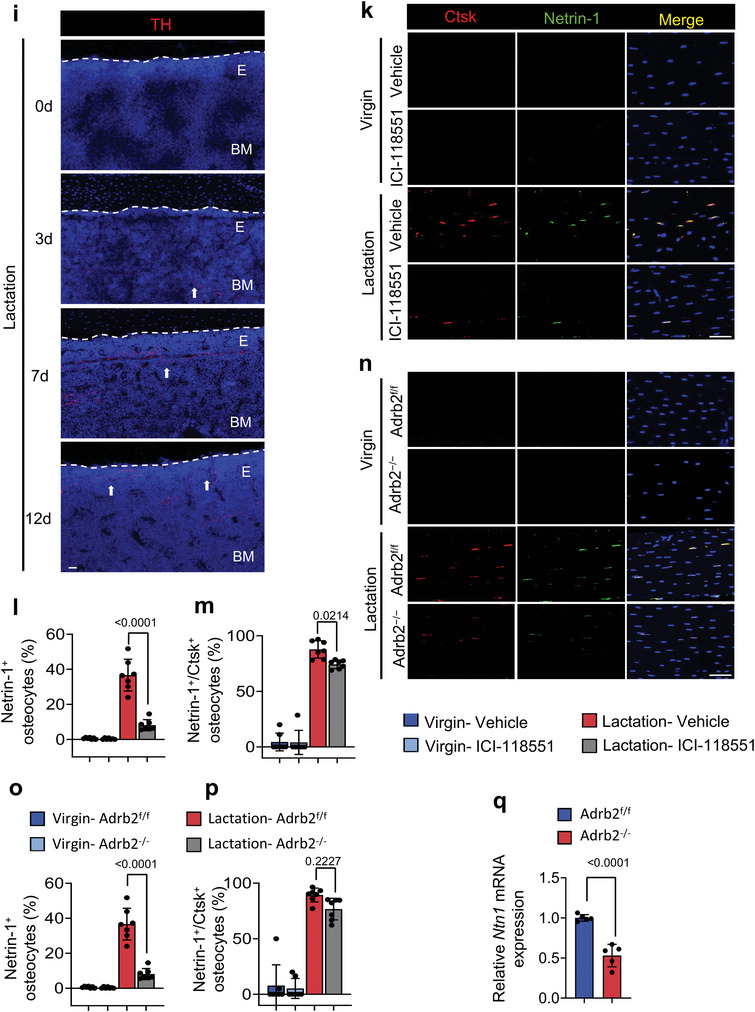

We next immunostained femoral bone sections of lactating mice and found that the extent of TH^+^ sympathetic nerve fibers progressively increased in the endosteal area from day 3 of lactation to day 12 (Figure 6i,j). We have shown that osteoclasts secrete netrin‐1 to induce sensory innervation,^[^ ^36^ ^]^ which likely explains how TH^+^ sympathetic innervation is abundant near the endosteum of the cortical bone. We further examined whether osteocytes also secrete netrin‐1 during lactation. Co‐immunofluorescent staining of Ctsk and netrin‐1 demonstrated that 80% of Ctsk^+^ osteocytes were also positive for netrin‐1 in the cortical bone of lactating mice, but injection of ICI‐118551 significantly reduces netrin‐1^+^ and netrin‐1^+^/Ctsk^+^ osteocytes in lactating mice compared to vehicle‐treated lactating mice (Figure 6k–m). Furthermore, the increase of netrin‐1^+^ osteocytes in cortical bone was significantly diminished in Adrb2*^−/−^* lactating mice relative to their Adrb2^f/f^ littermate controls, though the percentage of netrin‐1^+^/Ctsk^+^ osteocytes was not significantly changed in the knockout mice (Figure 6n–p). RT‐qPCR also showed that the expression of *Ntn1* was significantly decreased in Adrb2*^−/−^* mice compared to their Adrb2^f/f^ littermates during lactation (Figure 6q). Together, these results suggest that netrin‐1 promotes sympathetic nerve innervation to the endosteum of cortical bone.

### 2.7 Depletion of TH^+^ Sympathetic Nerves Ameliorate Osteocyte‐Mediated Perilacunar Bone Resorption in Lactating Mice

To validate that sympathetic innervation of the endosteum of cortical bone is the critical step by which osteocytes promote bone resorption, we prepared thermosensitive hydrogel Pluronic F127 with slow‐release of oxidopamine (6‐OHDA), a selective sympathetic nerve toxin that specifically depletes sympathetic nerves, and guanethidine, an inhibitor of NE release from the sympathetic nerve ending. The cumulative release testing confirmed the efficiency of sustained release of 30% F127 (Figure S3a, Supporting Information). F127+6‐OHDA, F127+guanethidine, or F127+vehicle was injected into the mice femur (**Figure** **7**a). We harvested the femurs 9 days after injection and found that the NE concentration was significantly lower in mice injected with F127+6‐OHDA or F127+guanethidine compared to the F127+vehicle treatment control group (Figure 7b). Also, we compared both ipsilateral and contralateral femurs of the lactating mice that accepted F127+6‐OHDA injection to confirm the accuracy of sympathetic nerve depletion by immunofluorescence. The TH^+^ sympathetic innervation is diminished significantly in ipsilateral compared to contralateral femurs. (Figure S3b, Supporting Information). Next, we immunostained femur sections and found that the extent of TH^+^ sympathetic nerves was markedly greater in the endosteal area of the lactating mice injected with F127+vehicle or F127+guanethidine compared to those injected with F127+6‐OHDA (Figure 7c).

Figure 7TH^+^ sympathetic nerve fibers are essential for osteocyte lacunar bone resorption. a) Schematic diagram illustrating the procedure of intra‐femoral injection of vehicle, F127/vehicle, F127/6‐OHDA, and F127/guanethidine in mice after 3 days of lactation. b) NE levels in tibial cortical bone chips determined by ELISA in virgin and lactating mice with different treatments. n = 5. c) Immunofluorescence staining of TH (red) in the endosteum area in femoral bone marrow. E, endosteum; BM, bone marrow. n = 5. d–h) Representative micro‐computed tomography (µCT) images (d) and quantitative analysis of open porosity (Po.op) (e), total porosity (Po.tot) (f), cortical thickness (Cor.Th) (g) of femoral cortical bone and trabecular bone fraction (BV/TV) (h). Scale bar, 1 mm. n = 5. i–o) Representative images for tartrate‐resistant acid phosphatase (TRAP) staining (i), immunofluorescence staining of Ctsk (green) (k) and Alcian blue staining (m) on femoral cortical bone of virgin and lactating mice with different treatments, quantification of the percentage of TRAP^+^ osteocytes (j) and Ctsk^+^ osteocytes (l), and calculation of osteocyte lacuna area (n) and osteocyte lacuna perimeter (o). Scale bar, 40 µm. n = 5. Data are presented as mean ± SEM. Statistical significance was determined by one‐way repeated measures ANOVA with Dunnet post hoc test.

**Figure:**
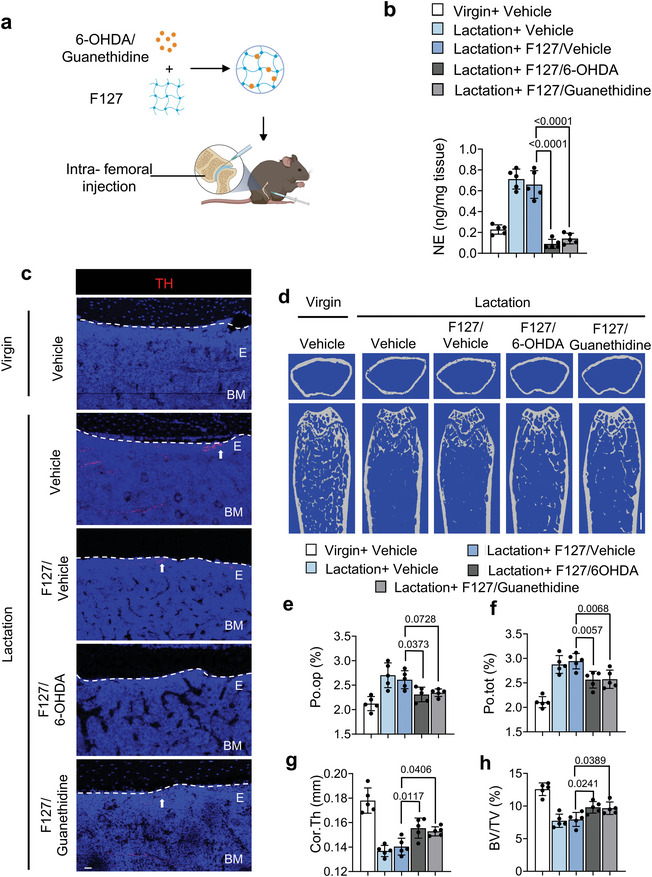

**Figure:**
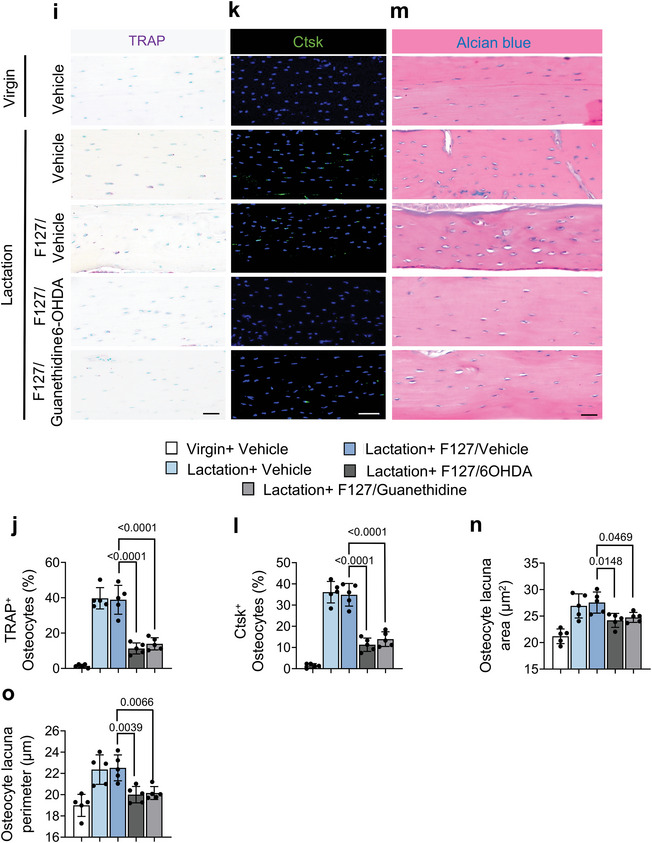

We next analyzed the effects of reducing TH^+^ sympathetic innervation and NE concentrations in the cortical bone. By µCT analysis, we found that the percentages of Po.op and Po.tot were both significantly lower in the F127+6‐OHDA‐treated lactating mice compared to those treated with F127+vehicle, though only the percent Po.tot was significantly lower in the F127+guanethidine‐treated group compared to the F127+vehicle group (Figure 7d–f). Further, we found that the Cor.Th and BV/TV were significantly higher in lactating mice injected with F127+6‐OHDA or F127+guanethidine compared to those treated with F127+vehicle (Figure 7g,h).

To test whether the changes in the bone parameters were associated with osteocyte perilacunar resorption, we performed TRAP staining and immunostaining for Ctsk in femur sections from the treated mice. We found that the percentage of TRAP^+^ and Ctsk^+^ osteocytes in cortical bone was significantly diminished in lactating mice injected with F127+6‐OHDA or F127+guanethidine compared to the F127+vehicle‐treated control groups (Figure 7i–l). Likewise, the osteocyte lacunar area and the osteocyte lacunar perimeter were both significantly lower in the lactating mice treated with F127+6‐OHDA compared to the F127+vehicle controls, though the lactating mice treated with F127+guanethidine showed a nonsignificant difference for both parameters compared to the F127+vehicle controls (Figure 7m–o). Together, these results suggest that the elimination of TH^+^ sympathetic innervation or inhibition of NE release in cortical bone reduces osteocyte‐mediated perilacunar resorption.

## 3 Discussion

**Figure 8:**
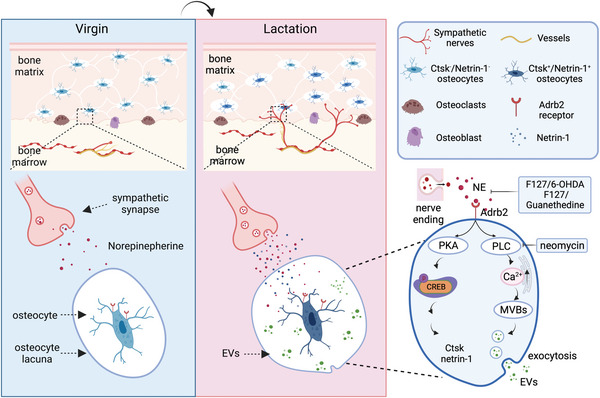
Summary diagram of SNS‐mediated regulation of lactation‐related osteocyte perilacunar resorption. During lactation, NE levels increase in the cortical bone. In response, osteocytes increase Ca^2+^ signaling to promote the secretion of bone resorptive EVs and Ctsk to degrade the perilacunar area bone. In parallel, NE signaling drives the phosphorylation of CREB to promote Ctsk and netrin‐1 expression, which enhances SNS tone signal, thus leading to an increased innervation of TH^+^ sympathetic nerves in the endosteum in a feedback loop manner.

In the current study, mouse lactation was used as a model to investigate the role of innervation in the regulation of osteocytic bone resorption. We found that sympathetic outflow was elevated during lactation, leading to NE‐mediated stimulation of *β*2 adrenergic receptor signaling in both osteocytes and osteoblasts. As a result, osteocyte release of EVs was induced for their bone resorption, and osteoblast differentiation was reduced (**Figure** **8**). The function of elevated sympathetic activity in both induction of osteocyte resorption and reduction of osteoblast differentiation is to compensate for the Ca^2+^ and energy needs for lactation. Interestingly, the enlarged lacunae that result from osteocyte‐mediated resorption are largely found at the inner layers of the cortical bone that are adjacent to the endosteum and quite evenly spread along the length of the long bone during lactation. Similarly, an increase of porosity at the endosteal side of cortical bone was also observed in humans during aging.^[^ ^11^, ^37^ ^]^ Physiologically, resorption of the endosteum of cortical bone does not reduce the bone's mechanical supporting property as much as resorption of periosteum cortical bone or uniform resorption.^[^ ^38^ ^]^ Interestingly, we found that osteocytes also secreted netrin‐1 to induce sympathetic nerve sprouting towards cortical bone during lactation, which supports the observation that osteocytic bone resorption occurs at the inner layer of cortical bone in our current study. Bone is the largest endocrine organ and cortical bone accounts for approximately 80% of skeletal mass.^[^ ^38^, ^39^, ^40^ ^]^ The primary function of bone is to provide mechanical support for bodily locomotion, as well as provide an important reservoir for calcium and other mineral stores.^[^ ^1^, ^41^ ^]^ Apparently, the CNS possesses the capability to enable precise remodeling of the skeleton to maintain the proper balance between sufficient mechanical support and healthy mineral metabolism.

Skeletal interoception maintains bone homeostasis by modulation of sympathetic outflow.^[^ ^14^ ^]^ In particular, an increase of COX2 expression in osteoblasts results in the production of PGE2, which in turn activates its receptor EP4 signaling through nerve terminals in the bone to promot TH expression and NE synthesis to drive sympathetic outflow from the hypothalamus.^[^ ^19^, ^20^ ^]^ Interestingly, osteoblast‐derived PGE2 reduces sympathetic activity in the hypothalamus, but knockout of the *Cox2* gene specifically in osteocytes does not affect the sympathetic activity in the hypothalamus.^[^ ^19^ ^]^ Therefore, osteocyte‐derived PGE2 is not involved in PGE2‐regulated skeletal interoception, and the up‐regulation of NE synthesis and sympathetic activity is likely not through a PGE2^+^ ascending nerve pathway originating from the osteocytes. As prolactin receptor is expressed in the hypothalamus and is inducible during lactation,^[^ ^42^, ^43^ ^]^ it is possible that prolactin receptor signaling is involved in the upregulation of sympathetic activity that occurs during osteocyte‐driven lacunar bone resorption. Further studies are needed to explore that possibility.

Aquatic vertebrates do not have osteocytes nor does their skeleton undergo remodeling as they do not bear gravity.^[^ ^44^ ^]^ Osteocytes were developed in terrestrial vertebrates that evolved from aquatic vertebrates.^[^ ^45^ ^]^ Lactation is one of the primary features of mammals, and breastfeeding is a complex physiological process that involves many different organs and particular energy‐related metabolic outputs.^[^ ^46^ ^]^ In addition to hormonal regulation, sympathetic nervous system activity has been reported to play a role in the regulation of lactation.^[^ ^47^ ^]^ During breastfeeding, NE levels are increased in the white adipose tissue (WAT) and decreased in the brown adipose tissue (BAT).^[^ ^48^, ^49^ ^]^ Indeed, expression of *Ucp1* is decreased in BAT of lactating mice compared to virgin mice (Figure S1b, Supporting Information). Sympathetic innervation promotes the mobilization of the WAT, thereby providing systemic energy to the lactating mother.^[^ ^48^ ^]^ Similarly, an increase in sympathetic activity and spouting of TH^+^ nerve fibers in the endosteum and cortical bone induces osteocyte‐driven perilacunar bone resorption to mobilize calcium and other organic matter for the milk during lactation. These results suggest that the SNS is utilized in a concerted effort to modulate systemic energy and mineral stores to meet the needs of lactation. And it may be that prolactin acts as a hierarchal hormone to coordinate these needs through regulation of the SNS, with osteocyte‐driven bone resorption playing an important role in this coordinated response.

We found here that the distribution of TRAP^+^ osteocytes gradually extends from the endosteum to the periosteum as lactation proceeds. Importantly, the degree of NE‐induced perilacunar resorption and the expression of bone degradation‐related enzymes was partly abolished by inhibiting the release of NE from nerve endings or by eliminating sympathetic nerves in the endosteal region. Together with higher expression of netrin‐1 in osteocytes and sprouting of TH^+^ nerve fibers in the endosteum area and cortical bone, these results demonstrate that high sympathetic tone initiates a positive feedback loop between osteocytes and sympathetic nerves during lactation.

The mechanism of osteocyte‐directed lacunar bone resorption is different from that of osteoclast‐mediated bone resorption, although both are TRAP^+^ cells.^[^ ^6^, ^24^, ^25^ ^]^ Osteoclasts are polarized and attach to the bone surface to form a physical seal as they acidify the underlying area to create a resorption pit. In contrast, osteocytes promote resorption inside the bone.^[^ ^24^ ^]^ Therefore, the lacunar network and microenvironment generated by osteocytes are essential for bone resorption. We have shown that sensory innervation in the subchondral bone is induced in osteoarthritis by osteoclast‐derived netrin‐1 secretio.^[^ ^50^ ^]^ Interestingly, we found here that osteocytes also secrete netrin‐1 to induce the sprouting of sympathetic nerves in the cortical bone. Moreover, nearly 80% of Ctsk^+^ osteocytes are also netrin‐1‐positive after 12 days of lactation.

Nerve‐regulated internal bone resorption conceivably has advantages over hormonal regulation of bone surface resorption. Here, we show that NE released by sympathetic nerve terminals stimulates osteocytes to produce and release EVs that contain bone degradation proteins, which is known to be a Ca^2+^‐driven process.^[^ ^13^ ^]^ The lacunar resorption microenvironment created by osteocytes upon release of EVs is also Ca^2+^ signaling dependent, as we found that inhibition of Ca^2+^ signaling diminished the levels of bone degradation proteins in isolated EVs. Moreover, osteocyte‐specific conditional knockout of Adrb2 significantly reduced the number of Ctsk^+^ and netrin‐1^+^ osteocytes. Thus, during lactation, likely in order to meet the demands for additional energy and mineral metabolism, elevated sympathetic outflow activates Adrb2 and Ca^2+^ signaling in the osteocytes to induce the production and release of EVs for internal cortical bone reportion. At the same time pCREB signaling is activated in the osteocytes to promote the expression of netrin‐1, which further promotes sympathetic activity to increase this process in a feed‐forward loop, which fits with the gradual increase in NE content and nerve sprouting that we observed over the course of 12 days of lactation.

Additional studies will be needed to understand how this process is reversed and the bone is rebuilt after lactation ceases. It is possible that upon breast tissue involution after weaning there is a further interoceptive signal from the breast to the CNS and back to the skeleton that diminishes SNS tone and allows the bone lost during lactation to be restored. Insight into that process in addition to the new insight into the physiology of bone loss during lactation obtained here may allow us to apply such knowledge to better treat bone loss that occurs under pathological conditions, especially as we found that NE content was increased in the cortical bone after both OVX and glucocorticoid treatment.

## 4 Experimental Section

### Mouse Strains and Constructions

Mice were housed under normal conditions with 12:12 h light: dark cycle in a temperature‐controlled room with food and water provided ad libitum. All animals were maintained in the animal facility of The Johns Hopkins University School of Medicine (Baltimore, MD). The experimental protocol was reviewed and approved by the Institutional Animal Care and Use Committee of Johns Hopkins University. C57BL/6J (WT, stock no. 000664) and Dmp1‐Cre (stock no. 023047) mouse strains were purchased from the Jackson Laboratory (Bar Harbor, ME). The Adrb2 ^flox/flox^ mouse strain was obtained from Gerald Karsenty's group (Columbia University, New York, USA). *Dmp1‐Cre* mice were crossed with Adrb2^flox/flox^ mice. The offspring were intercrossed to generate the following genotypes: wild type (referred to as WT in the text), *Dmp1‐Cre* (mice expressing Cre recombinase driven by the *Dmp1* promoter), Adrb2^flox/flox^ (mice homozygous for the Adrb2 floxed allele were referred to as “Adrb2^f/f^” herein), *Dmp1‐cre*::Adrb2 ^flox/flox^ (mice with Adrb2 conditionally deleted in *Dmp1*‐positive lineage cells were referred to as “Adrb2^−/−^” in the article). The genotypes of the mice were determined by PCR analyses of genomic DNA isolated from mouse tails with the following primers: *DMP1‐Cre*: forward: 5’‐TTGCCTTTCTCTCCACAGGT‐3’, *DMP1‐Cre* Reverse: 5’‐CATGTCCATCAGGTTCTTGC‐3’; Adrb2^flox/flox^: forward: 5’‐AGCTGAGTGTGCAGGACGCA‐3’, reverse: 5’‐CGCTTCGTCCCGTTCCTGAGT ‐3’.

### Lactation Model and In Vivo Treatment

Lactation studies in female mice were performed as previously described.^[^ ^51^ ^]^ Briefly, 12‐week‐old C57BL/6, Adrb2 ^flox/flox^, and *Dmp1‐Cre*::Adrb2 ^flox/flox^ female mice were crossed with male mice. After parturition, the litter size was culled to 6 to 7 pups to normalize suckling intensities and calcium requirements for the offspring. Females were killed on the 12^th^ day of lactation and were approximately 16 weeks old. The virgin mice were also killed at 16 weeks as a control group. Propranolol (Sigma) was added into the drinking water at a concentration of 500 mg mL^−1^ until killing. Mice were randomly divided into four groups: i) Virgin+ Saline, ii)Virgin+ Propranolol, iii) Lactation+ Saline, iv) Lactation+ Propranolol.

To block the Adrb2 receptor, Age matched virgin mice and lactation mice were randomized to Vehicle and ICI‐118551 treatment group. Mice were injected intraperitoneally with Vehicle or ICI‐118551 at 1 mg kg^−1^ body weight.

Taking advantage of the sustained drug release character of hydrogel, a thermoresponsive, injectable 6‐OHDA/Guanethidine formulation was designed based on Pluronic F127 to diminish the NE in endosteum area. After mixing F127 and 6‐OHDA/Guanethidine at a 3:7 ratio, the resulting combination was used to inject into the femur. Mice were randomly divided into five groups: Virgin+Vehicle, Lactation+Vehicle, lactation+F127/Vehicle (combination of 30% F127 and 70% PBS), Lactation+F127/6‐OHDA (combination of 30% F127 and 70% 6‐OHDA, the dose of 6‐OHDA was 30 mg), Lactation +F127/ Guanethidine (combination of 30% F127 and 70% Guanethidine, the dose of Guanethidine was 6 mg). All mice received intra‐bone marrow injections in right femur at 3 days and sacrificed at 12 days after breastfeeding.

The drugs and compounds used in this study were as follows: Propranolol (Prop, Sigma‐Aldrich, 1576005); ICI‐118551 (Sigma‐Aldrich, 72795‐19‐8); norepinephrine (Sigma‐Aldrich, A7257); Pluronic F127 (Sigma‐Aldrich,P2443); 6‐Hydroxydopamine hydrochloride(6‐OHDA) (Sigma‐Aldrich,H3481); Guanethidine monosulfate (Sigma‐Aldrich,1301801); neomycin (Sigma‐Aldrich). Dosages and time courses were noted in the corresponding text and figure legends.

### Glucocorticoid‐Induced Bone Loss Model

Glucocorticoid‐induced cortical bone loss in mice was performed as previously described.^[^ ^52^ ^]^ Briefly, 12‐week‐old C57BL/6J mice was injected with methylprednisolone (10 mg m^−2^ day^−1^) for 4 weeks. Meeh's formula was used to calculate the body surface area (BSA), with a *k* constant of 9.82 for mouse body weight (in grams) to the two‐thirds power (BSA = kW^2/3^).

### Ovariectomy Model

12‐week‐old female C57BL/6 mice were randomized to OVX and sham group. The operation was performed as previously described.^[^ ^6^ ^]^ In short, the mice were anesthetized at 12 weeks of age, and the abdominal cavity was opened through an incision in the middle of the abdomen between the last coastal ridge and the thigh. The abdominal cavity was closed aseptically after removing both ovaries.

### µCT Analysis

Mice were killed by inhaling isoflurane and then perfused with PBS and 4% buffered formalin. The femurs of mice were dissected and fixed in 4% paraformaldehyde at 4 °C overnight. Analysis of µCT was executed by using a high‐resolution µCT scanner (SkyScan, 1275). The scanning procedure was settled as 65 kv for voltage and 153‐µA for current, with a resolution of 6.4 µm per pixel. To analyze the cortical bone and the metaphyseal trabecular bone parameters of the femurs, reconstruction software (NRecon, v1.6, SkyScan), data analysis software (CTAn, v1.9, SkyScan), and 3D model visualization software (CTVol, v2.0, SkyScan) were selected. Cross‐sectional images of the femur were created to exhibit 3D analyses of cortical bone and trabecular bone. The region of interest (ROI) of the trabecular bone was started at proximally 0.5 mm from the distal metaphyseal growth plate and was then extended proximally for an additional 0.5 mm of the femur's length. The trabecular bone volume fraction (BV/TV) was collected from the 3D analysis data and used to represent the trabecular bone parameters. The area between 10% of the femur's length distal to the distal metaphyseal growth plate and another 10% of the femur's length proximally was defined as the cortical bone ROI. The cortical thickness (Ct.Th), open porosity (Po.op), close porosity (Po.cl), total porosity (Po.tot), and tissue mineral density of cortical bone (TMD) were collected from the 3D analyses data and used to represent the cortical bone parameters. The trabecular bone volume fraction (BV/TV) from 3D analyses data were selected to represent the cortical parameters. Phantoms were used to calibrate tissue mineral density of cortical bone (TMD).

### Immunofluorescence and Histomorphometry

Cells were fixed in 4% paraformaldehyde for 30 min after treatment for immunofluorescence staining. In vitro treatment of NE and ICI‐118551 were described before. Briefly, primary osteocytes were treated with 50 µm NE, 100µm NE, 100µm NE+50µm ICI‐118551, and 100µm NE+100µm ICI‐118551 for 30 min and washed with cold PBS for 3 times. Then, cultured cells were incubated with primary antibody of rabbit Phospho‐CREB (Cell signaling, Ser133,9198, 1:200), and rabbit Tsg101 (Abcam, ab133586, 1:100) at 4 °C overnight. After thoroughly washing the cells with PBS, the corresponding secondary antibodies conjugated with fluorochrome and DAPI (Invitrogen, Thermo Fisher Scientific H3569,1:500) were applied to the cells for 1 h at room temperature. The Olympus DP72 microscope was used to observe and measure the cells (Olympus Scientific Solutions Americas Inc., Waltham, MA).

For histochemical and immune‐histological analysis, the protocols were reported in the previous study.^[^ ^53^ ^]^ In brief, the femurs were collected and fixed in 10% buffered formalin for 48 h at 4 °C. After 3 weeks of decalcification in 0.5 m ethylenediaminetetraacetic acid (pH 7.4) (Amresco), samples were embedded in paraffin or optimal cutting temperature (OCT) compound (Sakura Finetek, Torrance, CA). 4‐µm‐thick femur sections were processed at coronal orientation for TRAP (Sigma‐Aldrich) and Alcian blue staining using standard protocols as used before.^[^ ^6^ ^]^ 10‐ and 40‐µm‐thick femoral coronal‐oriented sections were harvested for immunofluorescent staining by standard protocol.^[^ ^54^ ^]^ Briefly, the slides were incubated with primary antibodies to rabbit Adrb2 (Abcam, ab182136, 1:100), goat Adrb1 (Abcam, ab3442, 1:100), rabbit Cathepsin K (Abcam, ab19027, 1:100), rabbit Tyrosine hydroxylase (Abcam, ab137869,1:100), chicken Netrin‐1(Abcam, ab39379, 1:100), and rabbit Osteocalcin (Origene, BP710, 1:100) at 4° overnight. The slides were then incubated at room temperature for 1 h with secondary antibodies conjugated with fluorochrome and DAPI (Invitrogen, Thermo Fisher Scientific H3569,1:500). The specimen images were observed and recorded by using an Olympus DP72 microscope (Olympus Scientific Solutions Americas Inc., Waltham, MA) and a Zeiss 780 confocal microscope (Zeiss, Oberkochen, Germany). In each section, the total number of positively stained cells was counted in the cortical area of the proximal epiphysis, mid‐diaphysis, and distal metaphysis. Each mouse in each group had five nonsequential sections counted and averaged.

### RT‐qPCR

The tibias of each group of mice were dissected. After removing soft tissue and scraping off periosteum, the epiphysis was cut off. By syringe, bone marrow cells were flushed out with cold PBS. Bones were cut into 1‐ to 2‐mm lengths. Total RNA was extracted from isolated cortical bone segments using the TRIzol reagent (Invitrogen, Thermo Fisher Scientific) according to the manufacturer's instructions. The High‐Capacity cDNA Reverse Transcription Kit DNA (4374966, Thermo Fisher Scientific) was then used to reverse transcribe the RNA into complementary DNA. Next, the QuantStudio 3 Real‐Time PCR System (Thermo Fisher Scientific) was used to perform RT‐qPCR with Fast SYBR Green Master Mix (4385610, Thermo Fisher Scientific). The 2^−ΔΔCT^ method was used to calculate the relative expression of each gene, and Gapdh was chosen as the internal control for normalization. The primers used for each gene were as follows: *α1‐AR* forward: 5′‐CAAGGCCTCAAGTCCGGCCT‐3′, reverse: 5′‐ CTCTCGAGAAAACTTGAGCAG‐3′; *α2‐AR* forward: 5′‐GTGACACTGACGCTGGTTTG‐3′, reverse:5′‐CCAGTAACCCATAACCTCGTTG‐3′, *β1‐AR* forward: 5′‐GTCATGGGATTGCTGGTGGT‐3′, reverse: 5′‐GCAAACTCTGGTAGCGAAAGG‐3′; *β2‐AR* forward: 5′‐GGGAACGACAGCGACTTCTT‐3′, reverse: 5′‐GCCAGGACGATAACCGACAT‐3′; *Ntn1* forward: 5′‐CAGCCTGATCCTTGCTCGG‐3′, reverse: 5’‐ GCGGGTTATTGAGGTCGGTG‐3′; *Ucp1* forward: 5’‐ CTGCCAGGACAGTACCCAAG‐3′, reverse: 5’‐TCAGCTGTTCAAAGCACACA‐3′; *Gapdh* forward: 5’‐GGGTGTGAACCACGAGAAAT‐3’, reverse: 5’‐CCTTCCACAATGCCAAAGTT‐3’.

### Norepinephrine ELISA

Tissues were prepared similarly to those for RT‐qPCR detection. Each group of mice was sacrificed and the tibias were dissected. Soft tissue was removed through scraping, and both epiphyses were cut off. Bone marrow cells were flushed out with cold PBS by syringe. Bones were cut and homogenized into powder. Expression of NE was measured by a high‐sensitivity NE ELISA kit (Cat#NOU39‐K01, Eagle Biosciences) according to manufacturer's protocol. The same weight of bone chips was used to quantify protein concentration and was used to normalize NE measurements.

### Primary Osteocyte Isolation, Culture, and Characterization

The protocol of serial digestions of primary osteocytes was modified.^[^ ^55^, ^56^ ^]^ In brief, 3‐month‐old male mice were sacrificed, and the femurs and tibias were dissected and transferred into *α*‐minimal essential medium (*α*‐MEM; Gibco) containing 1% Penicillin‐Streptomycin (PS; Gibco). After removing connective tissue and periosteum, both epiphyses were cut off. Bone marrow cells were flushed out with cold PBS by using a syringe and then cut into ≈1 mm lengths and placed in Hank's balanced salt solution (HBSS). Bone fragments were incubated in the sequence of 30 min collagenase (2% of collagenase type I in culture medium, sigma, C9891, 1% FBS) for first 3 times, then, 25 min EDTA solution (5 mm EDTA, 0.1% BSA in PBS, pH 7.4), 25 min collagenase solution, 25 min EDTA solution, and 25 min collagenase solution for 4 times alternatively. The bone chips were washed with HBSS for 3 times after every collagenase digestion. Then, a tissue homogenizer was used to mince bone pieces in *α*‐MEM. The osteocyte‐enriched fractions were obtained and processed 3 times in 20 min EDTA solution, 20 min collagenase solution, and 20 min collagenase solution. All digestion steps were carried out in 6‐well plates with 2 mL solutions per mouse and rotated at 150 RPM with 5% CO_2_ in a 37°C cell incubator. The final 3 times digestions were osteocyte‐enriched suspension, which were collected and then plated in the collagen‐coated plates and cultured in *α*‐MEM with 5% fetal bovine serum (FBS), 5% fetal calf serum (FCS), and 1% PS. To characterize the purity of primary osteocytes, cells were resuspended in 100‐µL FACS buffer and incubated with anti‐ SOST antibody (AF1589, 1:100, R&D Systems) for 30 min at 4 °C. Cells from the same digestion batch were only incubated with secondary antibody served as negative controls.

### Purification of Extracellular Vesicles

For exosome purification from cell culture‐conditioned medium, primary osteocytes were cultured in media supplemented with 5% exosome‐depleted FBS and 5% exosome‐depleted FCS. To deplete bovine exosomes, the serums were centrifugation at 100 000 x g for 16 h. Conditioned medium from each respective group was collected every day and immediately frozen at −20 °C. Supernatants were collected from 3×24 h cell cultures and extracellular vesicles were purified by a standard differential centrifugation protocol.^[^ ^57^ ^]^ Shortly, culture supernatants were centrifuged at 2000 x g for 20 min to remove cell debris and dead cells (Beckman Coulter, L‐80). After centrifugation at 16 500 x g for 45 min, macrovesicles were pelleted and discarded. The supernatants were then centrifuged for 2 h at 4 °C at 100 000 x g. The pelleted micro‐vesicles were washed in PBS and then ultracentrifuged at 100 000 x g for 2 h at 4 °C. The pelleted micro‐vesicles were resolved in lysis buffer for the next western blot.

### Transmission Electron Microscopy

EV preparations (10 µL) were quickly rinsed in PBS for three times on carbon‐coated copper grids with 400 mesh (Electron Microscopy Sciences). After removing the extra solution and drying, the grids were measured via TEM (JEM 1010, JEOL, Japan) set to 80 kv to obtain TEM images. The EV size was measured by Image J.

### Western Blotting

Western blot analyses were performed on the protein of lysates from the primary osteocytes or EVs extracted from cell culture supernatants. For the cell lysate, the primary osteocytes were plated into 6‐well plates after sequential digestions. When the confluence of cells reached 70%, the culture supernatant was changed into FBS‐free medium for 12 h. NE was applied into the FBS‐free medium the next day, and primary osteocytes were harvested after 30 min of NE treatment. RIPA buffer (89900, Thermo Fisher Scientific) with protease inhibitors (78442, Thermo Fisher Scientific) was added for protein extraction. Purified vesicles were prepared as previously described.^[^ ^57^ ^]^ To obtain the proteins from extracellular vesicles, RIPA buffer containing protease inhibitors was added for each group of vesicles. The lysates were separated by sodium dodecyl sulfate‐polyacrylamide gel electrophoresis (SDS‐PAGE), and then blocked with 5% non‐fat dry milk at room temperature for 1 h. The primary antibody for rabbit Phospho‐CREB (Cell signaling, Ser133,9198, 1:1000), CREB (Cell Signaling Thechnology,1:1000), rabbit Tsg101(Abcam, ab133586, 1:1000), rabbit Alix (Millipore Sigma, ABC40, 1:1000), rabbit Cathepsin K (Abcam, ab19027, 1:1000), and GAPDH (Cell Signaling, 2118, 1:1000) was applied for incubation at 4  °C overnight, then incubated with HRP‐conjugated secondary antibodies (Cell Signaling Technology) at room temperature for 1 h. The proteins were detected by SuperSignal West Femto Maximum Sensitivity Substrate (Thermo Fisher Scientific).

### Treatment of Primary Osteocytes

Primary osteocytes were treated with norepinephrine (A7257, Sigma), ICI‐118551 (1458009, Sigma) at concentrations of 50 or 100 µm and neomycin (1458009, Sigma) at 10 mm.

### Intracellular Calcium Assay

The calcium level in osteocytes was determined using a calcium assay kit (ab102505; Abcam) according to the manufacturer's instructions. Calcium concentrations (mm) were calculated using a standard curve that was generated.

### In Vitro Hydrogel Cumulative Release Study

The dialysis method was used to investigate the in vitro drug release behaviors of Rhodamine‐loaded hydrogels (pH 7.4). The overdose of Rhodamine was mixed with 20% or 30% F127 and stirred at 100 rpm for 2 h at 4 °C. The unloaded Rhodamine was removed after washing 3 times with cold PBS. Then, the Rhodamine loaded hydrogel was placed in a dialysis bag (MW: 2.5 kDa) and kept in 100 mL PBS at 37°C incubator with stirring. At predetermined time intervals (0, 12, 24, 36,48,72, 96, 120, 144, 168, 192, and 216 h), 3 mL of the dissolution medium was taken out and replaced with the same volume of fresh PBS. The amount of Rhodamine was measured via UV–vis spectrum.

### Single‐Cell RNA‐Sequence (scRNA‐seq) Analysis

The Johns Hopkins School of Medicine for Genomics and Bioinformatics (Core facility) prepared single‐cell libraries, and the quality of cDNA samples was assessed using an Agilent 2100 Expert High Sensitivity DNA Assay. CellRanger (version 5.0.1) was used to process the sequencing reads and align them to the mm10 reference genome. Prior to concatenation and batch correction, each individual sample was subjected to quality control in Seurat (version4.0.3). Ribosomal and mitochondrial genes, as well as MALAT1, were excluded from the list of features.

Then, clusters were annotated according to marker gene expression, differential expression analysis on annotated clusters, and SingleCellNet classification. To classify single cells in the data, a top‐pair Random Forest classifier was used in SingleCellNet on a well‐annotated reference dataset of mouse bone marrow (The Tabula Muris Consortium). Next, subpopulations of interest (i.e., preosteoblast and mature osteoblast) were assigned to subsets of the larger dataset, and the analysis steps outlined above were repeated for each subpopulation. Plots of gene expression were created in Seurat using the Dot Plot, Violin Plot, and Feature Plot functions.

### Statistical Analysis

All error bars were s.d. Data presented as mean ± s.d. Unpaired, two‐tailed Student's *t*‐tests were used for comparisons between two groups and one‐way analysis of variance (ANOVA) with Dunnett post hoc test or two‐way analysis of variance (ANOVA) with Tuckey post hoc test multiple comparisons. All data demonstrated a normal distribution and similar variation between groups. All data showed a normal distribution with similar variation between groups. All inclusion/exclusion criteria were predetermined, and no samples or animals were excluded. The sample size was not determined using any statistical method. The experiments were all random. During the experiments and outcome evaluation, the investigators were not blinded to allocation.

## Conflict of Interest

The authors declare no conflict of interest.

## Supporting information

Supporting Information

## Data Availability

The data that support the findings of this study are available from the corresponding author upon reasonable request.
