# Supplementary material for: Sympathetic Innervation Regulates Osteocyte‐Mediated Cortical Bone Resorption during Lactation
Source: Adv Sci (Weinh). 2023 Apr 26;10(18):2207602. doi: 10.1002/advs.202207602 (PMC10288263; doi:10.1002/advs.202207602)
Supplement: Supplementary file 1 — Supporting Information [file ADVS-10-2207602-s001.pdf]

ADVANCED SCIENCE
Open Access

Supporting Information

for *Adv. Sci.*, DOI 10.1002/advs.202207602

Sympathetic Innervation Regulates Osteocyte-Mediated Cortical Bone Resorption during Lactation

*Qiaoyue Guo, Ningrong Chen, Cheng Qian, Cheng Qi, Kathleen Noller, Mei Wan, Xiaonan Liu, Weixin Zhang, Patrick Cahan and Xu Cao**

Fig. S1

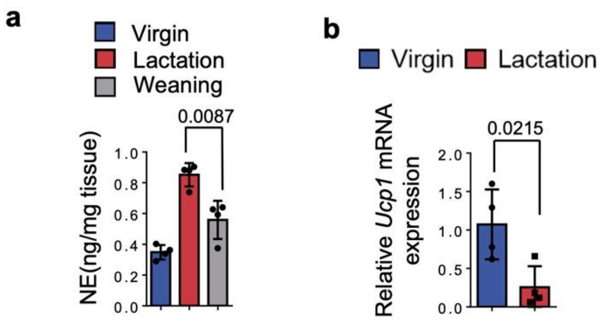

Fig. S1: Sympathetic tone signals were increased during lactation with decrease of *Ucp1* mRNA expression in subscapular BAT. **(a)** Norepinephrine levels of isolated cortical bone chips in virgin, lactating, and weaning mice by ELISA. n = 4 mice in each group. **(b)** mRNA expression of *Ucp1* by RT-qPCR in subscapular BAT of WT virgin and lactating mice. n= 4. Data are presented as mean ± SEM. Statistical significance was determined by one-way ANOVA with Dunnet post hoc test (a) and two-tailed Student's *t*-tests (b).

Fig. S2

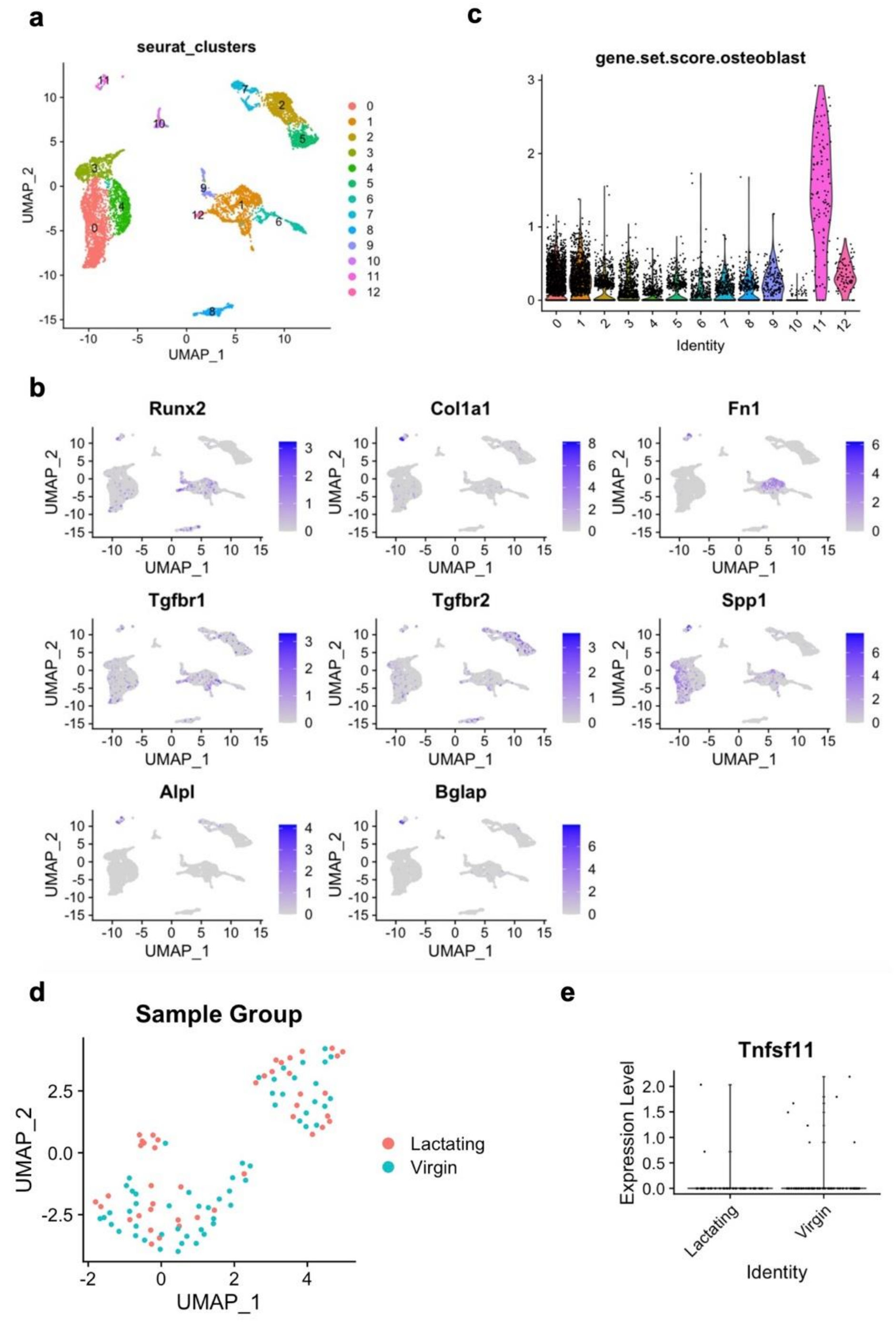

Fig. S2: Bioinformatics analysis of scRNA-seq of femur from lactating and virgin mice. (a) Long bones were subjected to serial collagenase/EDTA digestions (see methods), and cells from fractions 8, 9, and 10 were collected for flow cytometry. Live cells were sorted followed by single-cell RNA-seq library construction. Cell-clustering was performed in each group. n=8 per group. **(b)** Feature plots showing the expression

of known osteoblast lineage marker of each cluster. **(c)** Violin plots of osteoblast lineage marker genes expression in cell populations. **(d)** Feature plots showing the cells distribution in virgin and lactation groups in cluster 11. **(e)** Violin plots of *Tnfs11* gene expression in cluster 11.

Fig.S3

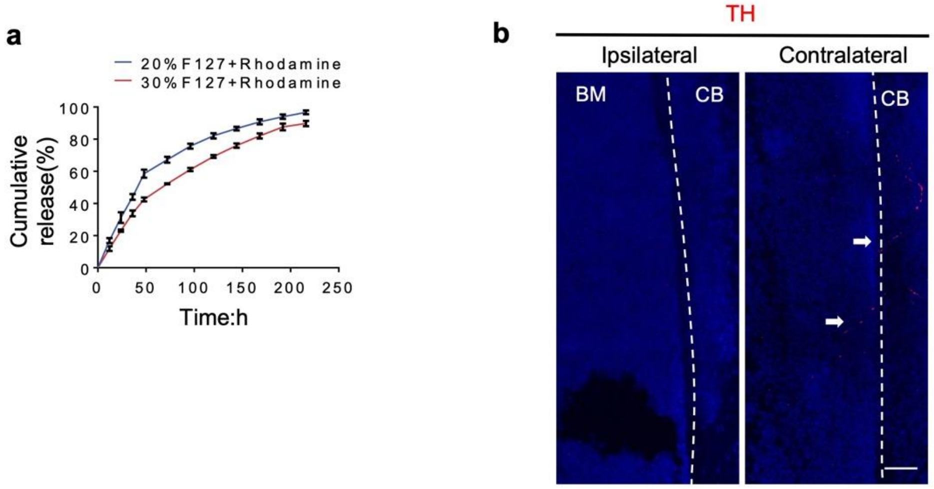

Fig. S3: 6-OHDA loaded F127 hydrogel depleted local sympathetic nerves in femur. (a) Release behavior of 20% and 30% F127 hydrogel. **(b)**Immunofluorescence staining of TH (red) in the endosteum area in femur bone marrow. CB, cortical bone; BM, bone marrow. n = 3 mice.
